# Intracardiac Echocardiography-Guided Percutaneous Aspiration of Tricuspid Vegetations

**DOI:** 10.1016/j.jscai.2023.101291

**Published:** 2024-01-18

**Authors:** Zach Rozenbaum

**Affiliations:** Department of Cardiology, Tulane University, New Orleans, Louisiana

**Keywords:** aspiration devices, infective endocarditis, intracardiac echocardiography, tricuspid vegetation

An intracardiac echocardiography-guided percutaneous aspiration of tricuspid vegetation was performed using a manual aspiration device. This precluded the need for an anesthesia team, an echocardiography team, and a perfusionist (Figure 1; Supplementary Video).

**Figure 1:**
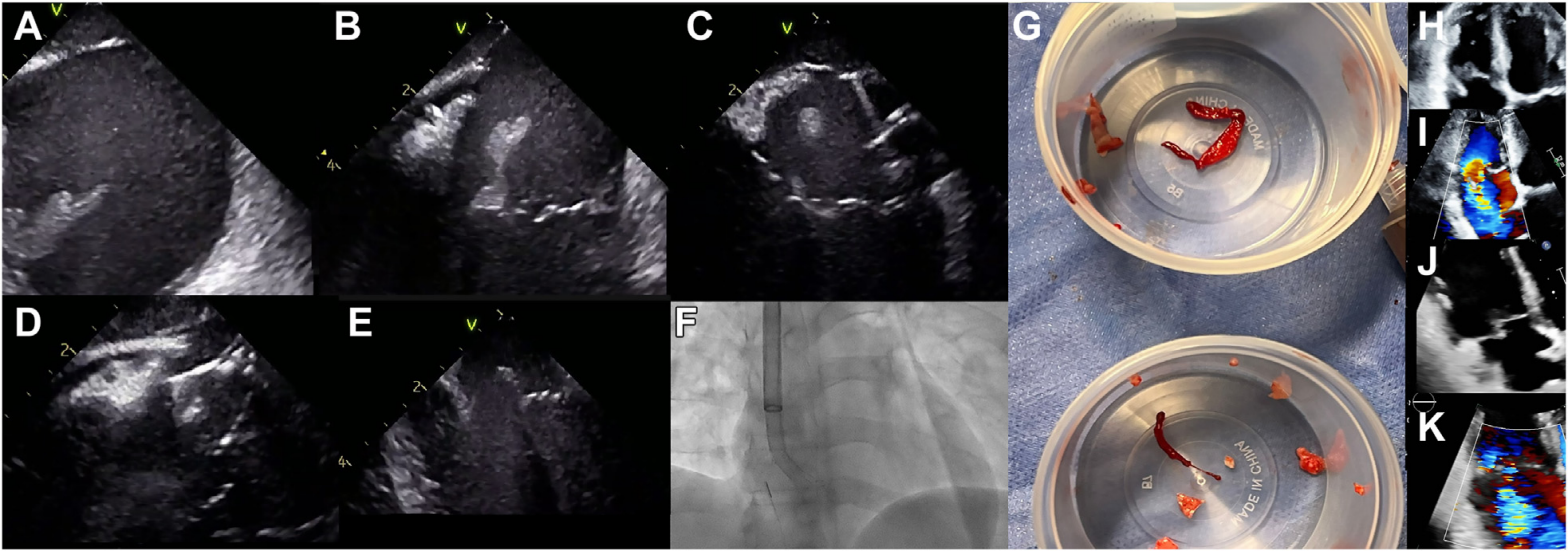
**Intracardiac echocardiography-guided percutaneous aspiration of tricuspid vegetations.** (**A, B**) Vegetation on the tricuspid valve. (**C**) Engaging with the cannula. (**D**) The vegetation is seen inside the funnel-shaped tip. (**E**) The leaflet of the tricuspid valve undamaged and free of vegetations. (**F**) An AlphaVac (AngioDynamics) cannula 26F sheath from the internal jugular vein to the right atrium and an intracardiac echocardiography catheter from the common femoral vein. (**G**) The aspirated material. (**H**) Vegetation on the tricuspid valve before aspiration. (**I**) Severe tricuspid regurgitation before aspiration. (**J**) The tricuspid valve after aspiration. (**K**) Moderate tricuspid regurgitation after aspiration.

## Supplementary material

Supplemental Video 1 Video clip of intracardiac echocardiography-guided percutaneous aspiration of tricuspid vegetations.

